# Polymorphisms of *NFκB1* and *IκBα* and Their Synergistic Effect on Nasopharyngeal Carcinoma Susceptibility

**DOI:** 10.1155/2015/362542

**Published:** 2015-06-16

**Authors:** Yehua Liu, Fuman Qiu, Lei Yang, Rongrong Yang, Xiaorong Yang, Dongsheng Huang, Wenxiang Fang, Lisha Zhang, Qingping Jiang, Lan Zhang, Yifeng Zhou, Jiachun Lu

**Affiliations:** ^1^The State Key Lab of Respiratory Disease, Collaborative Innovation Center for Environmental Toxicity, The Institute for Chemical Carcinogenesis, Guangzhou Medical University, 195 Dongfengxi Road, Guangzhou 510182, China; ^2^Department of Genetics, Collaborative Innovation Center for Environmental Toxicity, Medical College of Soochow University, 199 Renai Road, Suzhou 215123, China

## Abstract

Nasopharyngeal carcinoma (NPC) is a multifactoral and polygenic disease with high prevalence in Southeast Asia and Southern China. Environmental factors and genetic susceptibility play important roles in NPC pathogenesis. In the present study, we tested the hypothesis that single nucleotide polymorphisms (SNPs) in nuclear factor-kappa B (*NFκB*) and its inhibitor (*IκBα*) conferred consistent risks for NPC. Four putatively functional SNPs (*NFκB1*: rs28362491del>ins ATTG; *NFκB2*: rs12769316G>A; *IκBα*: rs2233406C>T and rs696G>A) were analyzed to evaluate their associations with NPC risk in total 1590 NPC cases and 1979 cancer-free controls. We found that the rs28362491 insATTG variants (ins/del + ins/ins) in *NFκB1* conferred an increased risk of NPC (odds ratio [OR] = 1.30, 95% confidence interval [CI] = 1.09–1.55, and *P* = 2.80 × 10^−3^) compared with the del/del homozygous genotype. The rs696AA variant in *IκBα* had an increased risk of NPC (OR = 1.41, 95% CI = 1.20–1.66, and *P* = 2.28 × 10^−5^) by decreasing *IκBα* expression due to the modulation of microRNA hsa-miR-449a. Furthermore, both adverse genotypes of *NFκB/IκBα* and their interaction also exerted an increased risk on NPC. Taken together, Our findings indicated that genetic variants in *NFκB1* (rs28362491del>ins ATTG) and *IκBα* (rs696G>A) and their synergistic effect might contribute to NPC predisposition.

## 1. Introduction

Nasopharyngeal carcinoma (NPC) is a malignancy of the head and neck that originates from the epithelial lining of the nasopharynx [1]. There were an estimated 84,400 incident cases of NPC and 51,600 deaths in 2008, representing about 0.7% of the global cancer burden [2]. NPC is rare in most parts of the world but is a leading malignancy in Southeast Asia and Southern China, with high incidence rate (40 per 100,000 person-years) [3, 4]. This distinctively geographic and ethnic distribution of NPC indicates that NPC is a malignancy with complex etiology involving both genetic and environmental factors [5].

Accumulating researches have revealed several well-established risk factors for NPC, such as Epstein-Barr virus (EBV) infection [6], certain dietary factors [7], and family history of cancer [8]. Studies have demonstrated that EBV is involved in direct carcinogenesis by triggering various cellular responses including the activation of inflammation [9, 10]. As a crucial inflammatory mediator, nuclear factor kappa-B (*NFκB*) and its endogenous inhibitors* NFκBI* (*IκB*) provide a critical mechanistic link between inflammation and tumor [11–14]. It has been reported that many signal transduction pathways, originating from a wide multifarious cellular stimuli, converge on the* NFκB/IκB* complex playing an essential role in cell angiogenesis, cell adhesion, proliferation, antiapoptosis, and repressing immune response [15]. Furthermore, the abnormalities of* NFκB* signaling pathway provide the cells with the production of growth factors as well as resistance to apoptotic and genotoxic insults, contributing to multiple carcinogenesis processes including tumor initiation, promotion, invasion, and metastasis [16, 17].

*NFκB1* and* NFκB2* are two major forms of the* NFκB* family in human [18], and they can be inactivated by the most common protein of I*κ*B family, NFkB inhibitor *α* (*IκBα*) [19]. Since* NFκB* is responsible for the regulation of many other genes in disease progression, variants in the genes coding for the* NFκB* and* IκB* proteins could be potentially related to disease development. Previous studies have identified several single nucleotide polymorphisms (SNPs) in* NFκB1*/*NFκB2* and* IκBα* to be associated with a great variety of diseases including inflammatory disorder and cancer [20–23]. However, mechanisms behind how specific polymorphisms of these various genes associate with NPC are still unclearly known. So we hypothesized that the SNPs in* NFκB*/*IκBα* genes may influence the NPC susceptibility.

In the present study, we analyzed the associations between four putatively functional SNPs (rs28362491del>ins ATTG in* NFκB1*; rs12769316G>A in* NFκB2*; rs2233406C>T and rs696 G>A in* IκBα*) and NPC risk in 906 NPC patients and 1072 age and sex frequency-matched controls in southern Chinese, and then validated the remarkable findings with 684 NPC patients and 907 controls in eastern Chinese. Biochemical assays were further performed to identify the biological effects of these polymorphisms.

## 2. Material and Methods

### 2.1. Study Subjects

Two independent hospital-based case-control studies including a southern Chinese population as a discovery set and an eastern Chinese population as a validation set were previously described briefly [24]. In the discovery set, 906 cases and 1072 cancer-free controls were recruited from April 2002 to June 2010 in Guangzhou city. In the validation set, 684 NPC and 907 healthy controls were consecutively recruited from March 2001 to May 2009 in Suzhou city. After their provision of written informed consent, each participant was scheduled for an interview to provide information on smoking status, and other factors with a structured questionnaire and to donate 5 mL peripheral blood. The definitions of the smoking status, pack-years smoked, alcohol use, and the family history of cancer have been described elsewhere [25–27]. Moreover, the EBV infection status and tumor stages of cases were obtained from the medical records. The study was approved by the institutional review boards of Guangzhou Medical University and Soochow University.

### 2.2. SNP Selection and Genotyping

Several SNPs located at* NFκB* or* IκBα* gene have been identified in previous reports. Among them, four polymorphisms [i.e., rs28362491del>ins ATTG of* NFκB1*; rs12769316 G>A of* NFκB2*; rs2233406C>T and rs696G>A of* IκBα*] were putatively functional and reported to be associated with various human diseases [21, 22, 28–31]. Furthermore, no other SNPs except these four polymorphisms located in predicted 3000 bp promoter region, coding region, and 3′-untranslated region (3′-UTR) of* NFκB* or* IκBα* gene were common with minor allele frequency (MAF) > 5% in Chinese based on HapMap public database. Therefore, these four polymorphisms were selected in our study.

The genomic DNA of each subject was extracted from 2 mL whole blood using the DNA Blood Mini Kit (Tiangen, China) according to the manufacturer's instructions and the final concentrations of all DNA samples were normalized to 20 ng/ul with a good purity (OD_260_/OD_280_ = 1.8~2.0). TaqMan allelic discrimination assay, performing in the ABI PRISM 7500 Sequence Detection Systems (Applied Biosystems, Foster City, CA), was used to detect genotypes of the chosen SNPs. The genotypes were automatically determined by Sequence Detection Systems software 2.0.1 (Applied Biosystems; Supplementary Figure S1 in Supplementary Material available online at http://dx.doi.org/10.1155/2015/362542). Primers and probes were designed by Primer Express 3.0 software (Applied Biosystems) and synthesized by Shanghai GeneCore Biotechnologies (Shanghai, China) as shown in Supplementary Table S1. Approximately 10% of the samples were also randomly selected for a blinded regenotyping and 60 samples for sequencing, and the results were in 100% agreement (Supplementary Figure S1).

Because the biological effect of the rs28362491 del>ins ATTG variants has been evaluated elsewhere [20], here we focused on the functional effect of rs696 G>A polymorphism on NPC risk.

### 2.3. Construction of Reporter Plasmids

**Figure 1:**
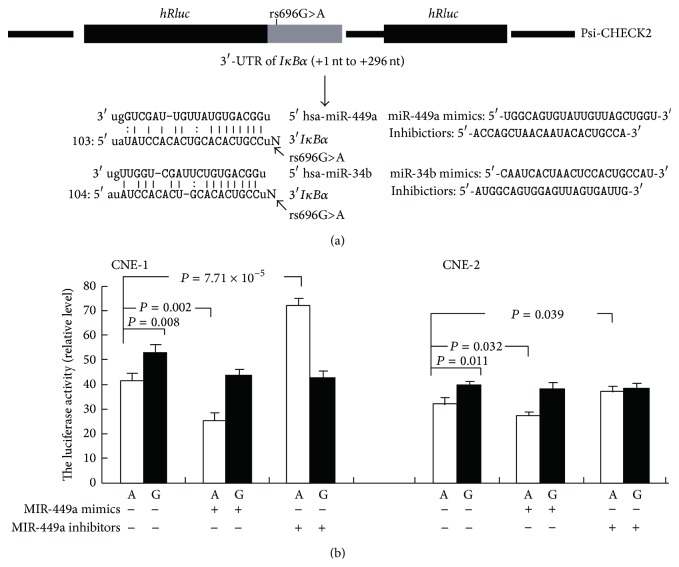
Effects of the rs696G>A polymorphism and treatment with microRNAs on* IκBα* transcriptional activity in different cell lines. (a). Schematic of the reporter gene construct with a 296 bp 3′-UTR of* IκBα* (+1 nt to 296 nt downstream to the translation stop site TGA) including rs696G>A polymorphism and a putative target site of miR-449a and miR-34b highly conserved in the* IκBα* mRNA 3′-UTR. (b). Luciferase expression of the two constructs in CNE-1 and CNE-2 cells. The renilla luciferase activity of each construct was normalized against the internal control of firefly luciferase. Columns, mean from three independent experiments; bars, SD; and Student's *t* test were used to test the differences in the expression levels of different constructs.

As a significant association was later observed for rs696G>A and NPC risk, we then constructed two reporter plasmids containing rs696 G or A allele to determine whether this polymorphism had any effect on its gene expression. The rs696G allele reporter was constructed by amplifying the 296 bp 3′-UTR of* IκBα* (+1 nt to +296 nt downstream to the translation stop site TGA) from subjects with homozygous rs696GG genotype using the forward primer: 5′-CCG ctcgag CGC AAA GGG GCT GAA AGA-3′ and reverse primer: 5′-ATA AGA AT gcggccgc ATA AAA TGT GGT CCT TCC ATG-3′, including the* XhoI* and* NotI* restriction sites. The amplified fragments and Psi-CHECK2 basic vector with renilla and firefly luciferase gene sequences (Promega, Madison, WI, USA) were then cocleaved by using* XhoI* and* NotI* (New England, BioLabs) and then ligated by T4 DNA ligase (New England, BioLabs) to product rs696G allele reporter gene (Figure 1(a)). The rs696A allele reporter gene was then obtained from the “G” construct by site-directed mutagenesis using the Quick-Change site-directed mutagenesis kit (Stratagene, La Jolla, CA, USA). All reporter constructs were sequenced to confirm the sequence, orientation and integrity of each insert.

### 2.4. RNA Interference, Transient Transfections and Luciferase Assays

Two human NPC cell lines CNE-1 and CNE-2 purchased from Cell Bank of Type Culture Collection of Chinese Academy of Sciences (Shanghai Institute of Cell Biology, Chinese Academy of Sciences) were conducted* in vitro* luciferase assays described previously [25, 26]. Because the bioinformatics analysis (http://www.targetscan.org/ and http://microrna.sanger.ac.uk/) showed that the rs696G>A would change the binding of the microRNA miR-449a and miR-34b, we then executed the RNA interference assay to show their effect interacted with this polymorphism. The cells were cultured in RPMI 1640 medium (Gibco-BRL, Gaithersburg, MD) with 10% fetal bovine serum (Gibco-BRL) and penicillin (100 units/mL)/streptomycin (100 *μ*g/mL) at a 37°C in the presence of 5% CO_2_. CNE-1 and CNE-2 were seeded onto 24-well plates with 1 × 10^5^cells per well and cultured for 24 h. The cells were then transiently transfected with 1.5 *μ*g of reporter plasmids (G or A allele) alone or co-transfected with or without microRNA mimics or inhibitors synthesized by GenePharma Co (Shanghai, China) using Lipofectamine 2000 according to the manufacturer's protocol (Invitrogen, Carlsbad, CA). The activities of* IκBα*-Psi-CHECK2 reporter with renilla luciferase and the internal standard with firefly luciferase was then measured by a Dual-Luciferase Reporter Assay System (Promega, Madison, WI, USA) in followed 14–16 hours. Independent triplicate experiments were done for each plasmid construct.

### 2.5. Statistical Analysis

Chi-square test or Student's *t* test was used to assess the difference in the distributions of demographic characteristics and genotypes between cases and controls. The association between each SNP and cancer risk was estimated using an unconditional logistic regression model with adjustments for surrounding factors. A multiplicative interaction was suggested to detect the possible gene-environment or gene-gene interactions [25]. Homogeneity test was performed with Breslow-Day test. The statistical power was calculated by the PS Software [32]. Student's *t* test was also used to examine the difference in levels of luciferase reporter gene expression between different constructs. All tests were two-sided by using the SAS software (version 9.3; SAS Institute, Cary, NC, USA) and *P* < 0.05 was considered statistically significant.

## 3. Results

### 3.1. *NFκB/IκBα* Genotypes and NPC Risk

The distributions of demographic characteristics for all participants were described previously [24] and presented in Supplementary Table S2. Briefly, no significant deviations were observed in age, sex, and family history of cancer between cases and controls. However, other factors including smoking, drinking status, and EBV infection were significantly different (*P* < 0.05 for all). Furthermore, the homogeneity test revealed that the frequency distributions of drinking sand EBV infection status were not homogeneous between the two populations (*P* < 0.05), reflecting slightly different lifestyle among two populations.

The observed genotype frequencies of all SNPs were in agreement with the Hardy-Weinberg equilibrium in controls (*P* > 0.05 for all) as shown in Table 1. In discovery set, we found that the rs28362491ins variant genotypes (ins/ins + ins/del) of* NFκB1* conferred a 1.28-fold increased risk of NPC compared with del/del genotype (odds ratio [OR] = 1.28; 95% confidence interval [CI] = 1.01–1.63; *P* = 0.011) in a dominant genetic model, which is best fitted to criteria of the smallest AIC value. The individuals carrying rs696AA genotype exerted a 1.46-fold increased NPC risk compared to those with rs696G (GG + GA) genotypes (OR = 1.46; 95% CI = 1.17–1.82;* P* = 7.4 × 10^−4^) under a recessive genetic model. However, for other polymorphisms, no significant associations with NPC susceptibility were observed.

The above remarkable findings were confirmed from the validation set. As a consistent harmful role of* NFκB1* rs28362491del/ins and* IκBα* rs696G>A polymorphism for NPC risk, individuals carrying rs28362491ins variant genotypes had a 1.31-fold increased cancer risk compared with del/del genotype (OR = 1.31; 95% CI = 1.02–1.70; *P* = 0.009), and the rs696AA adverse genotype present a 1.38-fold risk of NPC compared to rs696 (GG + GA) genotypes (OR = 1.38; 95% CI = 1.09–1.75; *P* = 0.007). We then merged the two sets to increase the study power because the associations of the adverse genotype in the two datasets were homogeneous (*P* = 0.941 for rs28362491del>ins ATTG and *P* = 0.938 for rs696G>A). We found that the rs28362491ins variant genotypes of* NFκB1* had a 30% excess risk of NPC (OR = 1.30; 95% CI = 1.09–1.55;* P* = 2.80 × 10^−3^) compared to del/del genotype. Similarly, the rs696AA variant genotype conferred a 1.41-fold increased cancer risk (OR = 1.41; 95% CI = 1.20–1.66;* P* = 2.28 × 10^−5^).

We further explored the combined adverse genotypes of these two polymorphisms on the NPC risk. We defined rs28362491ins variant genotypes (ins/del + ins/ins) and rs696AA genotype as risk genotypes. The carriers of rs28362491del/del and rs696GG/AG have zero risk genotype, the carriers of ins/del (ins/ins) and rs696GG/AG or del/del and rs696AA have one risk genotype, and the ins/del (ins/ins) and rs696AA carriers have two risk genotypes. We found that the number of risk genotypes had consistently significant associations with NPC risk in the discovery set, the validation set, and the merged set. Compared to the zero risk genotype, the individuals carrying risk genotypes conferred an increased risk of NPC in a dose-dependent manner in the pooled populations (OR = 1.25; 95% CI = 1.02–1.52; *P* = 0.029 for one risk genotype; OR = 1.82; 95% CI = 1.44–2.31;* P* = 6.34 × 10^−7^ for two risk genotypes; *P*_trend_ = 2.98 × 10^−7^).

### 3.2. Stratification Analysis and* NFκB1*-*IκBα* Interaction on NPC Risk

As shown in Table 2, the significant dose-effect of number of adverse genotypes on NPC risk were observed in all the subgroups. In addition, the potential gene-gene interaction of rs28362491del/ins and rs696G>A polymorphism on the risk of NPC was also investigated. We found that the individuals carrying rs696AA variant genotype conferred a more prominent adverse role on the risk of NPC compared to those with rs696GG/AG genotypes, while accompanied with rs28362491del/ins unfavorable genotype (OR = 1.38; 95% CI = 1.09–1.74; *P* = 0.007) or with rs28362491ins/ins adverse variant (OR = 1.53; 95% CI = 1.17–2.02; *P* = 0.002). Moreover, a significant positive interaction between the variations of* NFκB1* and* IκBα* on NPC risk was also observed (*P* = 2.25 × 10^−6^, shown in Table 3).

### 3.3. Luciferase Activity Assay

As visualized in Figure 1(b), luciferase assays showed that the transcription activity of the reporter gene which integrated the* IκBα* 3′-UTR with rs696A allele was suppressed more efficiently than that with G allele both in CNE-1 and CNE-2 cell (*P* value is 0.008 and 0.011, resp.).

The miR-449a mimics could further reduce the reporter genes' activity with rs696A allele (*P* = 0.002), and the miR-449a inhibitor reversed and upregulated reporter genes' activity (*P* = 7.71 × 10^−5^). However, the miR-34b failed to exhibit any effect on the reporter genes either with rs696A or G allele (*P* > 0.05 for all, data not shown). Taken together, these results indicated that hsa-miR-449a but not hsa-miR-34b specially binds to rs696A allele of the* IκBα* 3′-UTR and thus suppresses the expression of the* IκBα* gene* in vitro*.

## 4. Discussion

In the present hospital-based retrospective study, we found that the rs28362491ins ATTG variants of* NFκB1* conferred an increased risk of NPC, and the rs696AA variant of* IκBα* contributed an increased risk of NPC by decreasing* IκBα* expression under the modulation of hsa-miR-449a but not hsa-miR-34b. Both unfavorable genotypes of* NFκB1* and* IκBα* and their interaction exerted an effect on increasing NPC risk. To the best of our knowledge, this is the first study to investigate the genetic variants in* NFκB1* and* IκBα* on the risk of NPC.

Various studies have demonstrated that* NFkB1* and* IκBα* play a critical role in complicated human pathologies by regulating downstream genes involved in the immune response, cell proliferation, apoptosis, and senescence in addition to tumorigenesis [33, 34]. As a vital role in LMP1-mediated signal transduction, upregulated expression or overactivation of* NFκB1* has been reported to promote the NPC initiation [35]. Meanwhile,* IκBα*, which functions to suppress the effect of* NFκB1*, has been elucidated to be inactive or downregulated during various stimuli induced* NFκB* activation progresses and, in consequence, loses its protective role for human disease [36, 37]. Taking into consideration of the vital function on carcinogenesis and tumor progression by* NFκB/IκBα* manipulations, whether variations within the* NFκB* and its inhibitory protein* IκBα* could potentially influence the function of* NFκB* and in turn facilitate tumor development were noteworthy.

Several evidences have been evaluated that* NFκB* and* IκBα* polymorphisms were associated with a series of cancer types including bladder cancer [38], colorectal cancer [39], and lung cancer [40]. Previous studies have provided the testification of rs28362491ins ATTG variants relatively increased the* NFκB1* gene expression and thus promoted the susceptibility of human disease [20, 41]. And another two studies of 479 gastric cancer cases and 880 controls in Japanese [42] and 1001 sporadic colorectal cancer patients and 1005 cancer-free controls in Chinese [22] also displayed harmful role of rs28362491ins variants for gastric cancer and CRC risk. Furthermore, rs696G>A polymorphism in the 3′-UTR of* IκBα* showed an increased risk of developing CRC in Chinese population [39]. In the current study, we received a consistent result as the rs28362491ins variants contributed an unfavorable effect on NPC susceptibility. Likewise, we also found that rs696G>A polymorphism in the 3′-UTR of* IκBα* gene conferred an increased risk of NPC. It is well known that microRNA could cause mRNA cleavage or translational suppression via imperfect binding to the 3′-UTR of target genes. According to the bioinformatics analysis, we found that the rs696G>A would change the potential bindings for the microRNAs miR-449a and miR-34b. We then performed a luciferase assay* in vitro* and the results indicated that the rs696A allele strengthened the binding capacity of miR-449a but not miR-34b, to the 3′-UTR of* IκBα* gene, which in turn inhibited the* IκBα* transcriptional activities. Correspondingly, recent literatures have identified the content that variations located in microRNA binding sites could affect miRNA-target recognition efficiency and gene expression and thus potentially to be associated with cancers [43]. This reconciles with our findings that the miR-449a could specially regulate the activities of* IκBα* genes with rs696A but not G allele and thus influence the risk of NPC.

We further analyzed the combined effect of the* NFκB1* and* IκBα* polymorphisms and their possible interaction on NPC risk. We found the adverse number of genotypes of* NFκB1*/*IκBα* offered an increased risk for NPC in a dose-dependent manner. Meanwhile, a significant interaction of* NFκB1* and* IκBα* variations on NPC risk was also observed. As previous biological mechanism indicated that rs696G>A SNP in* IκBα* could depress its expression; in contrast, rs28362491ins variants may relatively increase the* NFκB1* gene expression. It is reasonable about the fact that rs696G>A polymorphism might abolish the suppression of* IκBα* to* NFκB1* and upexpression* NFκB1* caused by rs28362491ins variants; these alterations in turn facilitated the carcinogenesis of NPC, which is potently supported our findings as gene-gene interaction of* NFκB1* and* IκBα* polymorphisms contributing a detrimental role on NPC risk.

As a hospital-based case-control study, some limitations in current study such as information bias would be ineluctable. However, with the fairly large sample size and two study populations, we have achieved high statistical powers (87.7% for* NFκB1* and 99.0% for* IκBα*) of the associations between* NFκB1/IκBα* polymorphism and NPC risk, and biological experiments also confirmed these significant associations. In addition, for the gene-gene interaction, we further analyzed the false-positive report probability (FPRP) and found that, under the assumption of a 0.0001 prior probability and a 1.50 prior OR as suggested by Wacholder et al. [44], the FPRP for the observed interaction of rs28362491ins variants and rs696A variant genotypes on NPC risk yielded a value of 0.027, which is lower than the preset FPRP-level criterion 0.20, indicating that our finding is noteworthy.

## 5. Conclusion

In conclusion, this preliminary study indicated that both* NFκB1* and* IκBα* polymorphisms were associated with NPC risk. Remarkable interaction between these two SNPs on the risk of NPC was also observed. These findings suggest that polymorphisms of* NFκB1* and* IκBα* may contribute a synergistic effect on NPC susceptibility in Chinese population. Validations with larger population-based studies in different ethnic groups and further biological assays are warranted to confirm our findings.

## Supplementary Material

Figure S1. Genotyping results of the selected SNPs in the NFκB and IκBα genes. (A). Genotyping of rs28362491del>ins ATTG in NFκB1, rs12769316G>A in NFκB2, rs2233406C>T and rs696G>A in IκBα detected by the TaqMan allelic discrimination assay. (B). The genotypes of the four SNPs were validated by direct sequencing and the results were all 100% concordant. Table S1. Primary information on the TaqMan assay of four SNPs in the NFκB and IκBα genes. Table S2. Frequency distributions of selected variables in NPC patients and controls. Briefly, only the factors including smoking, drinking status, and EBV status had significant different between cases and controls in the two study populations.

## Figures and Tables

**Table 1:** Distribution of genotypes of *NFκB*/*IκBα* and associations with the risk of NPC.

Genotypes/alleles	Discovery set			Validation set			Merged set		
	Case *n* (%)	Controls^a^ *n* (%)	Adjusted OR (95% CI)^b^	Case *n* (%)	Controls^a^ *n* (%)	Adjusted OR (95% CI)^b^	Case *n* (%)	Controls^a^ *n* (%)	Adjusted OR (95% CI)^b^
Total number of subjects	906	1072		684	907		1590	1979	
Total number of alleles	1812	2144		1368	1814		3180	3958	
*NFκB1*:									
rs28362491 del/ins									
del/del	152 (16.8)	224 (20.9)	1.00 (ref.)	117 (17.1)	195 (21.5)	1.00 (ref.)	269 (16.9)	419 (21.2)	1.00 (ref.)
del/ins	438 (48.3)	512 (47.8)	1.22 (0.94–1.56)	331 (48.4)	438 (48.3)	1.25 (0.95–1.64)	769 (49.4)	950 (48.0)	**1.24 (1.03–1.49)**
ins/ins	316 (34.9)	336 (31.3)	**1.39 (1.06–1.82)**	236 (34.5)	274 (30.2)	**1.42 (1.06–1.90)**	552 (34.7)	610 (30.8)	**1.41 (1.16–1.71)**
Trend test *P* value			**0.012**			**0.010**			**0.001**
Dominant model^c^			** **			** **			
ins/ins + del/ins versus del/del	754 (83.2)	848 (79.1)	**1.28 (1.01–1.63)**	567 (82.9)	712 (78.5)	**1.31 (1.02–1.70)**	1321 (83.1)	1560 (78.8)	**1.30 (1.09–1.55)**
*NFκB2*:									
rs12769316 G>A									
GG	485 (53.5)	599 (55.9)	1.00 (ref.)						
AG	347 (38.3)	391 (36.5)	1.06 (0.87–1.29)						
AA	74 (8.2)	82 (7.6)	1.13 (0.80–1.60)						
Trend test *P* value			0.320						
*IκBα*:									
rs2233406 C>T									
CC	701 (77.4)	813 (75.8)	1.00 (ref.)						
TC	188 (20.7)	244 (22.8)	0.90 (0.72–1.13)						
TT	17 (1.9)	15 (1.4)	1.54 (0.74–3.19)						
Trend test *P* value			0.618						
*IκBα*:									
rs696 G>A									
GG	256 (28.3)	317 (29.6)	1.00 (ref.)	182 (26.6)	255 (28.1)	1.00 (ref.)	438 (27.5)	572 (28.9)	1.00 (ref.)
AG	415 (45.8)	540 (50.4)	0.94 (0.76–1.17)	318 (46.5)	463 (51.1)	0.94 (0.74–1.20)	733 (46.1)	1003 (50.7)	0.94 (0.80–1.11)
AA	235 (25.9)	215 (20.1)	**1.40 (1.08–1.82)**	184 (26.9)	189 (20.8)	**1.33 (1.01–1.76)**	419 (26.4)	404 (20.4)	**1.37 (1.13–1.65)**
Trend test *P* value			**0.021**			**0.035**			**0.003**
Recessive model^c^			** **						
AA versus GG + AA	235 (25.9)	215 (20.1)	**1.46 (1.17–1.82)**	184 (26.9)	188 (20.7)	**1.38 (1.09–1.75)**	419 (26.4)	403 (20.3)	**1.41 (1.20–1.66)**
Combined *NFκB1/IκBα* genotypes									
Number of risk genotypes^d^									
0	105 (11.6)	183 (17.1)	1.00 (ref.)	99 (14.5)	148 (16.3)	1.00 (ref.)	204 (12.8)	331 (16.7)	1.00 (ref.)
1	613 (67.7)	715 (66.7)	**1.48 (1.12–1.94)**	419 (61.3)	617 (68.0)	1.05 (0.76–1.34)	1032 (64.9)	1332 (67.3)	**1.25 (1.02–1.52)**
2	188 (20.7)	174 (16.2)	**1.93 (1.39–2.69)**	166 (24.3)	142 (15.7)	**1.71 (1.21–2.41)**	354 (22.2)	316 (16.0)	**1.82 (1.44–2.31)**
Trend test *P* value			**3.93 × **10^−4^			**9.42 × **10^−5^			**2.98 × **10^−7^

^a^The observed genotype frequencies among the control subjects were in agreement with the Hardy-Weinberg equilibrium (*p* ^2^ + 2*pq* + *q* ^2^ = 1) in the control subjects of both sets (*P* > 0.05 for all).

^b^Adjusted in a logistic regression model that included age, sex, smoking status, alcohol use, family history of cancer, and dataset.

^c^Akaike information criterion (AIC) value.

^d^Genotype combinations of two polymorphisms in the *NFκB1 *and *IκBα*: ins variant genotypes rs28362491 (ins/del + ins/ins) and rs696AA genotype are defined as risk genotypes.

**Table 2:** Stratification analysis of the number of risk genotypes in *NFκB1/IκBα* by selected variables in NPC patients and controls.

	Patients (*n* = 1590)			Controls (*n* = 1979)			Adjusted OR (95% CI)^a^			*P*_trend_^c^
	0^b^	1^b^	2^b^	0^b^	1^b^	2^b^	0^b^	1^b^	2^b^	
	*n* (%)	*n* (%)	*n* (%)	*n* (%)	*n* (%)	*n* (%)				
Age (years)										
<50	86 (13.0)	425 (64.2)	151 (22.8)	145 (17.0)	578 (67.6)	132 (15.4)	1.00 (ref.)	1.27 (0.94–1.72)	**1.88 (1.31–2.71)**	**3.47 × **10^−4^
≥50	118 (12.7)	607 (65.4)	203 (21.9)	186 (16.5)	754 (67.1)	184 (16.4)	1.00 (ref.)	1.25 (0.97–1.63)	**1.83 (1.34–2.51)**	**2.56 × **10^−4^
Sex										
Male	135 (11.9)	743 (65.7)	253 (22.3)	232 (16.5)	946 (67.4)	226 (16.1)	1.00 (ref.)	1.35 (1.07–1.71)	**1.98 (1.49–2.63)**	**1.57 × **10^−6^
Female	69 (15.0)	289 (63.0)	101 (22.0)	99 (17.2)	386 (67.1)	90 (15.7)	1.00 (ref.)	1.06 (0.77–1.56)	**1.60 (1.07–2.20)**	**1.32 × **10^−6^
Family history of cancer										
YES	12 (7.3)	106 (64.2)	47 (28.5)	27 (15.8)	120 (70.2)	24 (14.0)	1.00 (ref.)	1.74 (0.82–3.71)	**3.92 (1.63–9.39)**	**9.60 × **10^−4^
NO	192 (13.5)	926 (65.0)	307 (21.5)	304 (16.8)	1212 (67.0)	292 (16.2)	1.00 (ref.)	1.22 (1.00–1.50)	**1.68 (1.31–2.15)**	**2.41 × **10^−5^
Smoking status										
Ever	98 (11.5)	579 (67.7)	178 (20.8)	158 (16.9)	630 (67.2)	149 (15.9)	1.00 (ref.)	1.50 (1.13–1.99)	**1.95 (1.39–2.76)**	**1.33 × **10^−4^
Never	106 (14.4)	453 (61.6)	176 (24.0)	173 (16.6)	702 (67.4)	167 (16.0)	1.00 (ref.)	1.05 (0.80–1.38)	**1.70 (1.22–2.37)**	**8.41 × **10^−4^
Drinking status										
Ever	103 (13.2)	497 (63.8)	179 (23.0)	98 (16.2)	415 (68.5)	93 (15.3)	1.00 (ref.)	1.13 (0.83–1.53)	**1.80 (1.23–2.62)**	**1.36 × **10^−3^
Never	101 (12.4)	535 (66.0)	175 (21.6)	233 (17.0)	917 (66.8)	223 (16.2)	1.00 (ref.)	1.35 (1.04–1.74)	**1.81 (1.33–2.47)**	**1.31 × **10^−4^
EBV infection										
Positive	161 (13.0)	791 (63.8)	287 (23.2)	57 (17.0)	222 (66.1)	57 (16.9)	1.00 (ref.)	1.27 (0.91–1.79)	**1.74 (1.14–2.66)**	**8.91 × **10^−3^
Negative	43 (12.2)	241 (68.7)	67 (19.1)	274 (16.7)	1110 (67.6)	259 (15.8)	1.00 (ref.)	1.39 (0.97–1.98)	**1.69 (1.10–2.58)**	**0.017**
Stages										
I	8 (10.3)	52 (66.7)	18 (23.1)	331 (16.7)	1332 (67.3)	316 (16.0)	1.00 (ref.)	1.55 (0.80–3.43)	**2.35 (1.02–5.46)**	**0.039**
II	51 (12.1)	279 (66.3)	91 (21.6)				1.00 (ref.)	1.35 (0.98–1.88)	**1.81 (1.27–2.73)**	**8.58 × **10^−4^
III	90 (13.0)	451 (65.1)	152 (21.9)				1.00 (ref.)	1.25 (0.96–1.62)	**1.79 (1.32–2.44)**	**1.35 × **10^−4^
IV	58 (14.6)	239 (60.1)	101 (25.3)				1.00 (ref.)	1.03 (0.73–1.38)	**1.84 (1.28–2.64)**	**2.97 × **10^−4^

^a^Compared with zero risk genotype, ORs were adjusted in a logistic regression model that included age, sex, smoking status, drinking status, and family history of cancer.

^b^Genotype combinations of the two polymorphisms in the *NFκB1* and *IκBα*: ins variant genotypes rs28362491 (ins/del + ins/ins) and rs696AA genotype are defined as risk genotypes: i.e., the carriers of rs28362491 del/del and rs696 GG/AG have zero risk genotype; the carriers of ins/del (ins/ins) and rs696 GG/AG, or del/del and rs696AA have one risk genotype; and the ins/del (ins/ins) and rs696AA carriers have two risk genotypes.

^c^Trend test for NPC risk, with number of risk genotypes in each stratum.

**Table 3:** Interaction analysis between the variations of *NFκB1* and *IκBα* on NPC risk.

	Cases (*n* = 1590) *IκBα*: rs696 G>A		Controls (*n* = 1979) *IκBα*: rs696 G>A		Crude OR (95% CI)^a^	Adjusted OR (95% CI)^a^	*P*_inter_^b^
	GG + AG *n* (%)	AA *n* (%)	GG + AG *n* (%)	AA *n* (%)	AA versus GG + AG	AA versus GG + AG	
*NFκB1*:							
rs28362491 del/ins							
del/del	204 (75.8)	65 (24.2)	331 (79.0)	88 (21.0)	1.20 (0.83–1.73)	1.24 (0.85–1.80)	
del/ins	575 (74.8)	194 (25.2)	762 (80.2)	188 (19.8)	1.37 (1.09–1.72)	**1.38 (1.09–1.74)**	
ins/ins	392 (71.0)	160 (29.0)	482 (79.0)	124 (21.0)	1.54 (1.18–2.01)	**1.53 (1.17–2.02)**	
Combined genotypes							**2.25 × **10^−6^
del/ins + ins/ins	967 (73.2)	354 (26.8)	1244 (79.7)	316 (20.3)	1.44 (1.21–1.71)	**1.45 (1.22–1.74)**	

^a^ORs were adjusted for age, sex and smoking status, and alcohol use, family history of cancer in a logistic regression model.

^b^ *P* value of test for the multiplicative interaction between *rs696* G>A genotypes and rs28362491 del/ins genotypes on cancer risk in logistic regression models.
