# Supplementary material for: Polymorphisms of NFκB1 and IκBα and Their Synergistic Effect on Nasopharyngeal Carcinoma Susceptibility
Source: Biomed Res Int. 2015 Jun 16;2015:362542. doi: 10.1155/2015/362542 (PMC4486297; doi:10.1155/2015/362542)
Supplement: Supplementary file 1 — Figure S1. Genotyping results of the selected SNPs in the NFκB and IκBα genes. (A). Genotyping of rs28362491del>ins ATTG in NFκB1, rs12769316G>A in NFκB2, rs2233406C>T and rs696G>A in IκBα detected by the TaqMan allelic discrimination assay. (B). The genotypes of the four SNPs were validated by direct sequencing and the results were all 100% concordant. Table S1. Primary information on the TaqMan assay of four SNPs in the NFκB and IκBα genes. Table S2. Frequency distributions of selected variables in NPC patients and controls. Briefly, only the factors including smoking, drinking status, and EBV status had significant different between cases and controls in the two study populations. [file 362542.f1.pdf]

Figure S1

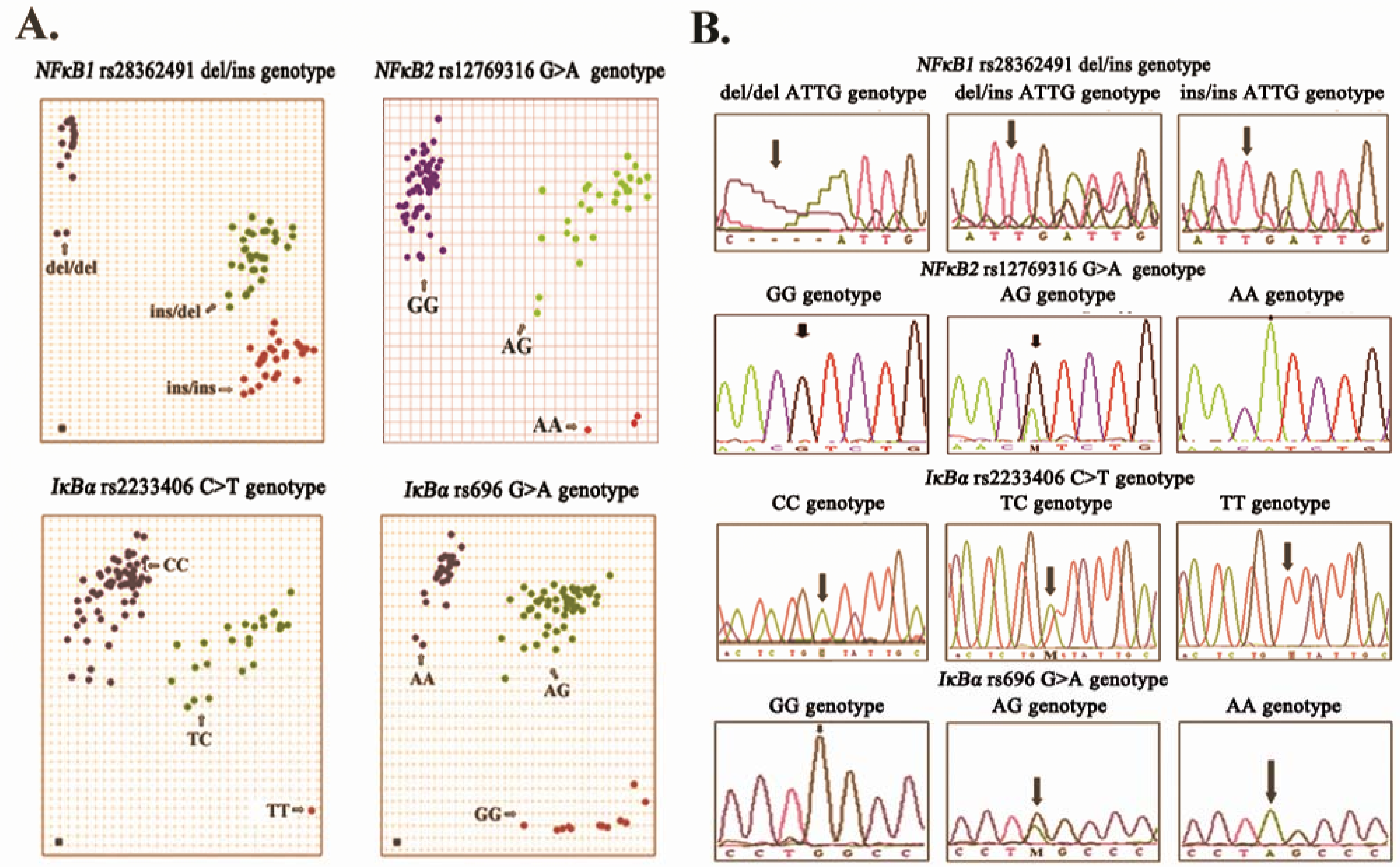
A.
NFκB1 rs28362491 del/ins genotype
del/del
ins/del
ins/ins
NFκB2 rs12769316 G>A genotype
GG
AG
AA
IκBα rs2233406 C>T genotype
CC
TC
TT
IκBα rs696 G>A genotype
AA
AG
GG
B.
NFκB1 rs28362491 del/ins genotype
del/del ATTG genotype
del/ins ATTG genotype
ins/ins ATTG genotype
NFκB2 rs12769316 G>A genotype
GG genotype
AG genotype
AA genotype
IκBα rs2233406 C>T genotype
CC genotype
TC genotype
TT genotype
IκBα rs696 G>A genotype
GG genotype
AG genotype
AA genotype

Supplementary Table S1. Primary information on the TaqMan assay of four SNPs in the *NFκB* and *IκBα* genes

Gene	Position and base change	Primers	Probes	Genotypes
NFκB1	rs28363491 ins/del ATTG	GCCTCCGTGCTGCCT (Forward)	P1: FAM-TTCCCCGACC**ATTG**G-MGB	del/del(blue)
		AGGGAAGCCCCCAGGAA (reverse)	P2: HEX-CCCGACC**----**ATTGATTG-MGB	ins/ins(red)
				del/ins(green)
NFκB2	rs12769316 G>A	GCCAGTGCAGGCCTGTGATTC (Forward)	P1: FAM-TAAAAAC**G**TCTGGGGC-MGB	G/G(blue)
		CCTGCCTGGAGTCCTCCTCCCT (reverse)	P2: HEX-AAC**A**TCTGGGGCAAGC-MGB	A/A(red)
				A/G(green)
IκBα	rs2233406 C>T	CGGGAGCACAATGTAGGTCA (Forward)	P1：FAM-CTACTCTG**C**TATTGCAA-MGB	C/C(blue)
		ATGTTTGTAGTGGTGGTTGTGGAT (reverse)	P2：HEX-CTACTCTG**T**TATTGCAAG-MGB	T/T(red)
				C/T(green)
IκBα	rs696 G>A	GCTGAAAGAACATGGACTTGTATATTTG (Forward)	P1：FAM-ACTGCCT**G**GCCCAA-MGB	G/G(blue)
		GGCTGATCCTACCACAATAAGACG (reverse)	P2：HEX-ACTGCCT**A**GCCCAAA-MGB	A/A(red)
				G/A(green)

[*] Bold nucleotide was the polymorphic site

[†] Three genotypes were indicated as three types of color dots in Supplementary Figure S1.

Supplementary Table S2. Frequency distributions of selected variables in NPC patients and controls

Variables	Discovery set (southern Chinese)			Validation set (eastern Chinese)			
	Case (n=906) n (%)	Control (n=1072) n (%)	*P* [a]	Case (n=684) n (%)	Control (n=907) n (%)	*P* [a]	*P* [b]
Age (years)							
< 50	396(43.7)	482(45.0)	0.576	266(38.9)	373(41.1)	0.368	0.758
≥ 50	510(56.3)	590(55.0)		418(61.1)	534(58.9)		
Sex							
Male	647(71.4)	763(71.2)	0.907	484(70.8)	641(70.7)	0.741	0.961
Female	259(28.6)	309(28.8)		200(29.5)	266(29.3)		
Family history of cancer							
Yes	88 (9.7)	83 (7.7)	0.120	77 (11.3)	88(9.7)	0.314	0.720
No	818(90.3)	989(92.3)		607(88.7)	819(90.3)		
Smoking status							
Ever	458(50.6)	481(44.9)	0.012	397(58.0)	456(50.3)	0.002	0.531
Never	448(49.4)	591(55.1)		287(42.0)	451(49.7)		
Drinking status							
Ever	440(48.6)	245(22.9)	<0.001	339(49.6)	361(39.8)	<0.001	<0.001
Never	466(51.4)	827(77.2)		345(50.4)	546(60.2)		
EBV Infection Status							
Positive	756(83.4)	194(18.1)	<0.001	483(70.6)	142(15.7)	<0.001	0.001
Negative	150(16.6)	878(81.9)		201(29.4)	765(84.3)		
Stages							
I	51 (5.7)			27 (3.9)			
II	244(26.9)			177(25.9)			
III	377(41.6)			316(46.2)			

IV	234(25.8)	164(24.0)

[a] *P* values for χ^2 test. [b] *P* values of homogeneity test for the frequency of case/controls within each valuable between two datasets.
